# Combination Effect of Phenylalanine‐Arginine‐Beta‐Naphthylamide and Curcumin on the Expression of the *mexY* Gene in Aminoglycoside‐Resistant Clinical Isolates of *Pseudomonas aeruginosa*

**DOI:** 10.1002/hsr2.70255

**Published:** 2024-12-10

**Authors:** Parisa Charkhi, Mohammad Reza Haghshenas, Bahman Mirzaei, Younes Khalili, Hamid Reza Goli

**Affiliations:** ^1^ Molecular and Cell Biology Research Centre, Faculty of Medicine Mazandaran University of Medical Sciences Sari Iran; ^2^ Department of Medical Microbiology and Virology, Faculty of Medicine Mazandaran University of Medical Sciences Sari Iran; ^3^ Department of Medical Microbiology and Virology, School of Medicine Zanjan University of Medical Sciences Zanjan Iran; ^4^ Immunology Research Center Tabriz University of Medical Sciences Tabriz Iran

**Keywords:** aminoglycoside resistance, efflux pump inhibitors, mexY, *Pseudomonas aeruginosa*

## Abstract

**Background and Aims:**

Overexpression of MexXY‐OprM efflux pump causes resistance to aminoglycosides in *Pseudomonas aeruginosa*. We aimed to investigate the relationship between resistance to aminoglycosides and the MexXY‐OprM expression level in *P. aeruginosa* clinical isolates without and after treatment with curcumin and/or phenylalanine‐arginine‐beta‐naphthylamide (PAβN) as the efflux pump inhibitors.

**Methods:**

We collected 100 clinical isolates from hospitalized patients. The minimum inhibitory concentrations of aminoglycosides were determined by the micro‐broth dilution method in the presence and absence of PAβN and/or curcumin. Then, real‐time PCR was used to determine the expression level of the MexXY‐OprM efflux pump.

**Results:**

In this study, 34%, 35%, 10%, 38%, 43%, 42%, and 39% of the clinical isolates were resistant to gentamicin, tobramycin, amikacin, netilmicin, spectinomycin, kanamycin, and streptomycin, respectively. Also, 45% of the isolates showed an overexpression of the *mexY* gene, while 31 (68.88%) isolates exhibited a 2–3‐fold overexpression, and 14 (31.11%) isolates had a more than threefold overexpression of the *mexY* gene. However, 4 (8.88%) isolates showed a ≥ 10‐fold overexpression of this gene. The combination of PAβN with spectinomycin, netilmicin, streptomycin, and kanamycin exhibited a reduced MIC range of these aminoglycosides in 93.02%, 86.8%, 76.9%, and 71.4% of resistant isolates, respectively. Additionally, all gentamicin‐, tobramycin‐, kanamycin‐, streptomycin‐, and netilmicin‐resistant isolates showed a decreased MIC range in combination with curcumin. The most synergistic effect of curcumin and PAβN was observed in combination with spectinomycin, while the least synergistic effect was detected with kanamycin.

**Conclusion:**

Curcumin can be a significant efflux inhibitor as an adjuvant in combination with aminoglycosides for successful treatment of patients infected by *P. aeruginosa* overexpressing the MexXY‐OprM efflux pump.

## 1 Background

*Pseudomonas aeruginosa* is a non‐fermenting gram‐negative organism and one of the most significant pathogens causing opportunistic infections in humans [1, 2]. This bacterium can live in all environments and is the cause of many problems in humans, such as burn and wound infections, sepsis, and chronic lung infections in patients with cystic fibrosis [1, 3]. *P. aeruginosa* belonged to the ESKAPE group of pathogens (*Enterococcus faecium*, *Staphylococcus aureus*, *Klebsiella pneumoniae*, *Acinetobacter baumannii*, *P. aeruginosa*, and *Enterobacter species*) [4]. However, these antibiotic‐resistant organisms are the major causes of nosocomial infections along with concerning therapeutic challenges [4]. The widespread drug resistant *P. aeruginosa* is one of the most significant concerns interfering with the treatment of infected patients [5]. Mechanisms involved in drug resistance include the production of metallo‐beta‐lactamases (MBLs), extended‐spectrum beta‐lactamases (ESBLs), and aminoglycoside‐modifying enzymes, along with reducing the entry of antibiotics into the cell and increasing the expression of efflux pumps [5]. Some antibiotics, such as beta‐lactams, aminoglycosides, and fluoroquinolones, are effective against this organism [5, 6]. However, continued prescription of these drugs as combination therapy, especially beta‐lactams and aminoglycosides, has increased the resistance to these antibiotics [7, 8].

Aminoglycosides are effective antibacterial agents that play a significant role in the treatment of *P. aeruginosa* infections, especially in cystic fibrosis (CF) patients [2, 9]. Antimicrobial resistance of *P. aeruginosa* against aminoglycosides has been reported since the 1960s [10]. However, resistance to these antibiotics is usually due to the production of chromosomal or plasmid‐encoded drug‐modifying enzymes, upregulation of efflux pumps, bacterial target (RNA) modification, and decreased drug influx [4, 7]. Another significant factor in the development of aminoglycoside resistance in *P. aeruginosa* is the overexpression of RND family efflux systems [11], as MexXY‐OprM is the only one to be able to efflux aminoglycosides [9, 12]. In addition, fluoroquinolones, aminoglycosides, tetracyclines, chloramphenicol, and erythromycin are also MexXY‐OprM substrates. This efflux system is not the same as MexAB‐OprM which is “constitutively” expressed [11]. MexXY‐OprM is produced at very low levels in wild type cells and is induced by some of its substrates [13].

The efflux pump inhibitors (EPIs) can be used to inhibit the MexXY‐OprM‐related aminoglycoside resistance in clinical isolates of *P. aeruginosa* [14]. The two EPIs used in the present study were curcumin and phenylalanine‐arginine beta‐naphthylamide (PAβN). PAβN is a broad‐spectrum competitive inhibitor of efflux pumps in *P. aeruginosa* [15]. However, the efflux pumps detect and remove it from the cell instead of the substrates, resulting in an increased concentration of the drugs [16]. On the other hand, curcumin, the active ingredient of the turmeric rhizome, is a polyphenolic compound with an efflux pump inhibitory effect, especially against multidrug‐resistant *P. aeruginosa*. The antibacterial effect of curcumin is due to the inhibition of FtsZ protein polymerization in bacterial cell wall [16]. Extensive research on this substance has shown its anti‐inflammatory, antiviral, antioxidant, anticancer, and antibacterial effects [17, 18]. Due to the significance of aminoglycosides as the treatment options for *P. aeruginosa*, the present study aimed to investigate the relationship between resistance to aminoglycosides and the MexXY‐OprM expression level in *P. aeruginosa* clinical isolates without and after treatment with curcumin and PAβN, as efflux pump inhibitors.

## 2 Methods

### 2.1 Ethical Approval Statement

This study was directed under the Declaration of Helsinki, although the written informed consent form was provided by the patients. The categorizing data of patients was kept secret. Moreover, this study was approved by the Iran National Committee for Ethics in Biomedical Research with the national ethical code IR. MAZUMS. REC.1397.368.

### 2.2 Sample Collection and Bacterial Identification

For this descriptive‐analytical study, 100 non‐repetitive clinical isolates of *P. aeruginosa* were estimated according to the followed formula, where *n* = sample size, *Z* = value of standard normal distribution (Z‐static) at 95% confidence level (*z* = 1.96), *p* = prevalence of aminoglycoside resistance in Iran (*p* = 55%) [19], and *d* = maximum error rate = 0.098.

$$n=\frac{z_{\alpha /2}^{2}\times p\left(1-P\right)}{d^{2}}$$

Collected from five therapeutic‐educational hospitals in Mazandaran province of Iran. These isolates were collected over 6 months (from September 2019 to February 2020) from different clinical samples and transferred to the microbiology department laboratory. All isolates were identified using standard conventional microbiology and biochemical techniques, including gram staining, pigment production on Mueller‐Hinton agar medium, non‐fermentation reaction, growth at 42°C, motility, citrate utilization, oxidase test, oxidation/fermentation (OF) test, and colony appearence [20]. Also, an API 20NE kit (Microgen TM, UK) was used to confirm the isolates. Confirmation of the *P. aeruginosa* isolates at the species level was accomplished by PCR of 16S rDNA‐based primer, as previously reported by Spilker et al. [21]. The pure isolates were then cultured in a TSB medium (Merck, Germany) containing 10% glycerol and stored in a freezer at −20°C.

### 2.3 Aminoglycoside Minimum Inhibitory Concentration (MIC)

The micro‐broth dilution method was used to determine the MIC of aminoglycosides (kanamycin, tobramycin, netilmicin, gentamicin, spectinomycin, amikacin, and streptomycin) (Sigma, Germany) against *P. aeruginosa* clinical isolates according to the CLSI guidelines [22]. First, 100 μL of cation‐adjusted Mueller‐Hinton broth (Sigma) was added to each well of 96‐well U‐shaped microplates. Then, the serial dilutions of the desired antibiotics were prepared in the wells. Finally, 105 cfu/well of the bacteria was added to all wells, except for negative control, which was contained the antibiotic and culture media. Additionally, a well was considered as positive control containing bacteria and broth medium. Then, the microplates were incubated at 37°C for 24 h, and the first concentration with no visual opacity was considered as MIC. According to the CLSI guideline, the isolates with a MIC ≥ 16 µg/mL were resistant to gentamicin and tobramycin, while ≥ 64 µg/mL and ≥ 32 µg/mL of the MICs were reported as resistance to amikacin and netilemicin, respectively [22]. Due to the lack of determination of MIC levels for kanamycin, spectinomycin, and streptomycin in CLSI, we considered a MIC ≥ 16 µg/mL as resistance to these drugs based on the defined ranges for gentamicin and tobramycin. The *P. aeruginosa* ATCC 27853 was used as a suseptible control strain in the micro‐broth dilution method.

### 2.4 MIC Determination in the Presence of Phenylalanine‐Arginine‐Beta‐Naphthylamide and Curcumin

All procedures were the same as for MIC determination. However, 50 µg/mL of the efflux pump inhibitors, including (PAβN (Sigma) and curcumin (Sigma)), were added to the reactions [8, 16]. These inhibitors were first tested separately with the antibiotics, and then were tested in combination. To combine the inhibitors, a 50 µg/mL concentration of each inhibitors was prepared in broth medium added to each well of the microplate. The MIC was also determined according to the CLSI guidelines. The isolates with a ≥four‐fold MIC reduction in the presence of inhibitors were considered as efflux pump overproducers [8, 16]. Additionally, the *P. aeruginosa* ATCC 27853 was used as the control in this test.

### 2.5 Total RNA Extraction and cDNA Synthesis

First, the RNAs of the clinical isolates were extracted by a total RNA extraction kit (Wizbiosolutions, South Korea) according to the manufacturer's instructions. Next, the extracted RNAs were treated with RNase‐free DNase I (Wizbiosolutions). Also, the RNA concentration and purity were determined by a NanoDrop spectrophotometer (ND‐1000, Wilmington, USA). Finally, the RNAs were stored at −70°C for further testing. After that, we used the Wizbiosolutions kit for cDNA synthesis, according to the manufacturer's instructions. Five micrograms of the extracted pure total RNA were added to the mixture to synthesize cDNA using M‐mulv reverse transcriptase and random hexamer primers (Wizbiosolutions). The synthesized cDNAs were stored at −20°C until use.

### 2.6 Quantitative Real‐Time PCR (qRT‒PCR)

Quantitative real‐time PCR was carried out using the SYBR premix EX TaqII, Tli RNaseH plus (Takara Bio Inc. Japan) in duplicate runs by an Applied Biosystems 7500 Real‐Time PCR machine (Thermo Fisher Scientific, USA). The specific primers were used to amplify the mexY gene (forward‐5’‐TCGCCCTATTCCTGCTG‐3’ and reverse‐5’‐AGTTCGCTGGTGATGCC‐3’) [23] and the rpoS housekeeping (normalizing) gene (forward‐5’‐CTCCCCGGGCAACTCCAAAAG‐3’ and reverse‐5’‐CGATCATCCGCTTCCGACCAG‐3’) [24]. The real‐time PCRs were performed at a final volume of 15 μL, including 7.5 μL of SYBR premix, 5 pmol of each primer, 300 ng of cDNA, and 5.5 μL of distilled water. The amplification condition for the rpoS gene included 95°C for 2 min and 40 cycles, including 20 s at 95°C, 30 s at 60°C, and 30 s at 72°C, while the real‐time PCR condition for the amplification of the mexY gene was as follows: 95°C for 2 min and 45 cycles containing 20 s at 95°C, 10 s at 60°C, and 30 s at 72°C. Moreover, a no template reaction (without cDNA) was used as a negative control, and the PAO1 wild‐type *P. aeruginosa* was used as the control strain in this test. A previously described relative quantification method (2^−ΔΔCt^ method) was used to calculate the gene expression levels of the clinical isolates compared to the PAO1 strain [25]. One isolate with an overexpression compared with the PAO1 strain was considered as a mexY hyperproducer [8]. Moreover, the Jfl10 strain (MexXY‐OprM overproducing *P. aeruginosa* mutant) was used as a positive control in real‐time PCR analysis [8].

### 2.7 Statistical Analysis

The data in the present study were analyzed by Statistical Package for the Social Sciences software version 22 and were compared by Chi‐square test. A *p* < 0.05 was considered statistically significant. All tests are repeated two times and the average of the results is reported.

## 3 Results

### 3.1 MIC Results of Aminoglycosides

The results of the broth microdilution test in the present study are shown in Table 1. According to these results, 43 isolates showed resistance to at least one aminoglycoside. Among these 43 isolates, 38 (88.37%), 35 (81.39%), 34 (79.06%), and 10 (23.25%) isolates were resistant to netilmicin, tobramycin, gentamicin, and amikacin, respectively. On the other hand, 43, 42, and 39 isolates showed a ≥ 16 μg/mL of MIC level against spectinomycin, kanamycin, and streptomycin, respectively, and were resistant to these aminoglycosides.

**Table 1:** The MIC results of aminoglycosides in 100 *P. aeruginosa* clinical isolates.

Antibiotics	Number of isolates with different MIC ranges (μg/mL)						
	≤ 4	8	16	32	64–128	256–1024	> 1024
**Gentamicin**	65	1	—	1	4	10	19
**Tobramycin**	64	1	1	3	9	22	—
**Amikacin**	76	5	4	5	5	5	—
**Kanamycin**	58	—	—	1	10	26	5
**Streptomycin**	58	3	9	8	14	7	1
**Spectinomycin**	57	—	1	—	3	31	8
**Netilmicin**	58	3	1	7	18	12	1

### 3.2 The Role of Efflux in Resistance to Aminoglycosides

The effect of efflux pump inhibitors on reducing the MIC of aminoglycosides against clinical isolates of aminoglycoside‐resistant *P. aeruginosa* is shown in Table 2. The combination of spectinomycin, netilmicin, streptomycin, and kanamycin with PAβN reduced the MIC of these aminoglycosides in 93.02%, 86.84%, 76.92%, and 71.42% of the resistant isolates, respectively. Additionally, the combination of amikacin, gentamicin, and tobramycin with this inhibitor showed a reduction in MIC range in 60%, 44.11%, and 57.14% of these resistant isolates, respectively. A more than threefold MIC reduction in combination with this inhibitor was detected in 10.52% of the netilmicin‐resistant and 10% of amikacin‐resistant isolates.

**Table 2:** Single and combined effects of efflux pump inhibitors on the aminoglycoside‐resistant isolates of *Pseudomonas aeruginosa*.

Inhibitors	Aminoglycosides	Number of resistant isolates	No. (%) of resistant isolates with reduced MIC	No. (%) of aminoglycoside‐resistant isolates with an unchanged or reduced MIC after treatment with efflux inhibitors				*p* value
				Unchanged	2‐fold	3‐fold	> 3‐fold	
**PAβN**	Gentamicin	34	15 (44.11)	19 (55.88)	12 (35.29)	—	3 (8.82)	0.09
	Tobramycin	35	20 (57.14)	15 (42.85)	16 (45.71)	2 (5.71)	2 (5.71)	0.07
	Amikacin	10	6 (60)	4 (40)	5 (50)	—	1 (10)	0.06
	Kanamycin	42	30 (71.42)	12 (28.57)	30 (71.42)	—	—	0.04
	Streptomycin	39	30 (76.92)	9 (23.07)	28 (71.79)	2 (5.12)	—	0.03
	Spectinomycin	43	40 (93.02)	3 (6.97)	29 (67.44)	8 (18.60)	3 (6.97)	0.00
	Netilmicin	38	33 (86.84)	5 (13.15)	24 (63.15)	5 (13.15)	4 (10.52)	0.01
**Curcumin**	Gentamicin	34	34 (100)	—	14 (41.17)	11 (32.35)	9 (26.47)	0.00
	Tobramycin	35	35 (100)	—	23 (65.71)	6 (17.14)	6 (17.14)	0.00
	Amikacin	10	6 (60)	4 (40)	3 (30)	—	3 (30)	0.06
	Kanamycin	42	42 (100)	—	30 (71.42)	8 (19.04)	4 (9.52)	0.00
	Streptomycin	39	39 (100)	—	17 (43.58)	11 (28.20)	11 (28.20)	0.00
	Spectinomycin	43	40 (93.02)	3 (6.97)	21 (48.83)	6 (13.95)	13 (30.23)	0.00
	Netilmicin	38	38 (100)	—	3 (7.89)	8 (21.05)	27 (71.05)	0.00
**Curcumin + PAβN**	Gentamicin	34	31 (91.17)	3 (8.82)	16 (47.05)	5 (14.70)	10 (29.41)	0.00
	Tobramycin	35	35 (100)	—	25 (71.42)	2 (5.71)	8 (22.85)	0.00
	Amikacin	10	9 (90)	1 (10)	7 (70)	1 (10)	1 (10)	0.01
	Kanamycin	42	32 (76.19)	10 (23.80)	32 (76.19)	—	—	0.03
	Streptomycin	39	37 (94.87)	2 (5.12)	23 (58.97)	8 (20.51)	6 (15.38)	0.00
	Spectinomycin	43	43 (100)	—	18 (41.86)	5 (11.62)	20 (46.51)	0.00
	Netilmicin	38	38 (100)	—	19 (50)	14 (36.84)	5 (13.15)	0.00

Abbreviations: MIC, minimum inhibitory concentration; PAβN, phenylalanine‐arginine‐beta‐naphthylamide.

On the other hand, all aminoglycoside‐resistant isolates showed a decreased MIC in combination with curcumin. However, the lowest synergistic effect was observed with amikacin, while 40% of the resistant isolates did not show any change in the MIC range. The highest reduction of MICs (more than threefold) was observed with the combination netilmicin/curcumin and spectinomycin/curcumin, in 71% and 30% of the isolates, respectively. Additionally, PAβN had a good synergistic effect with spectinomycin, netilmicin, and streptomycin, respectively. Moreover, all tobramycin‐, spectinomycin‐, and netilmicin‐resistant isolates showed a synergistic effect in combination with both studied inhibitors. The highest reduction of MICs (more than threefold) was found with the combination spectinomycin/curcumin and spectinomycin/PAβN (46.51%), while the least synergistic effect was observed with kanamycin. However, the effect of curcumin in reducing the MIC range of aminoglycosides was better than the combination of two inhibitors and the PAβN alone. Also, the synergistic effect of two inhibitors was better than the effect of PAβN alone.

**Figure 1:**
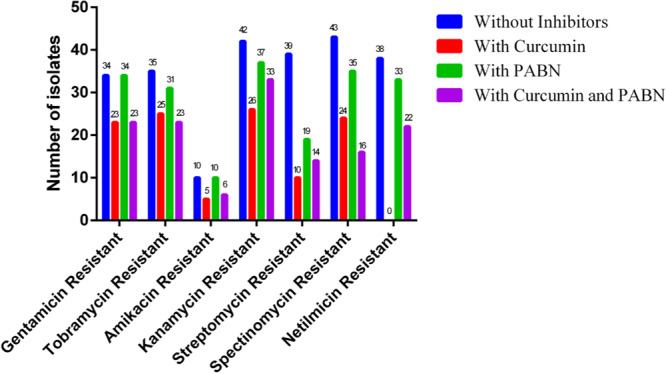
Susceptibility pattern of the aminoglycoside‐resistant isolates with and without the efflux pump inhibitors. The results of this figure show the number of resistant isolates before addition of the inhibitors and the number of resistant or susceptible isolates after treatment with the inhibitors.

Use of categorization resistance levels showed that when pump inhibitors were added, some resistant strains became intermediate‐resistant or even susceptible (Figure 1). Curcumin had the most synergistic effect with netilmicin, from which 30/38 (78.9%) resistant isolates showed susceptibility (*p* = 0.01). The same result (74.3% susceptibility) was observed in a combination of curcumin and streptomycin (*p* = 0.01). On the other hand, simultaneous synergism of both inhibitors (curcumin + PAβN) had the most effect with streptomycin and spectinomycin, from which 64.1% and 62.7% of the resistant isolates showed a susceptibility phenotype (*p* = 0.04), respectively.

### 3.3 *mexY* Expression and MIC Ranges of Aminoglycosides

Out of 100 clinical isolates in the present study, 45 isolates (45%) showed overexpression of the mexY gene compared to the PAO1 control strain. However, 31 and 14 isolates exhibited a two–three‐fold, and more than a three‐fold overexpression. Moreover, only four isolates showed a ≥ 10‐fold overexpression of MexXY‐OprM efflux pump compared to the PAO1 control strain. Table 3 shows the relation between the expression level of the MexXY‐OprM efflux operon and the MIC of aminoglycosides. There was a statistically significant corelation between the aminoglycoside MIC ranges and the expression level of the mexY gene in the present study (*p* < 0.05). For instance, 12 gentamicin‐non‐susceptible (resistant and intermediate‐resistant) isolates showed an increased level of the mexY gene expression, while just three gentamicin‐susceptible isolates exhibited the same feature. This ratio (non‐susceptible/susceptible) for tobramycin, amikacin, and netilmicin was 13/2, 6/9, and 15/0, respectively. If a MIC ≥ 16 μg/mL is considered as spectinomycin, kanamycin, and streptomycin resistance range, the ratios of nonsusceptible/susceptible isolates with overexpression were 15/0, 15/0, and 13/2, respectively. There was a significant relationship between the mexY overexpression and resistance to all aminoglycosides, except for amikacin, (*p* < 0.05).

**Table 3:** Relationship between the expression level of mexY gene and the MIC of aminoglycosides of 100 clinical isolates of *P. aeruginosa*.

Antibiotics	Number of mexY overproducer or nonoverproducer isolates considering different MIC ranges (μg/mL)														*p* value
	≤ 4		8		16		32		64–128		256–1024		> 1024		
	I	N	I	N	I	N	I	N	I	N	I	N	I	N	
**Gentamicin**	3	62	0	1	0	0	0	1	2	2	6	4	4	15	0.045
**Tobramycin**	2	62	1	0	0	1	0	3	6	3	6	16	0	0	0.041
**Amikacin**	4	72	3	2	2	2	3	2	1	4	2	3	0	0	0.035
**Kanamycin**	0	58	0	0	0	0	1	0	4	6	9	17	1	4	0.045
**Streptomycin**	1	57	1	2	3	6	2	6	4	10	4	3	0	1	0.043
**Spectinomycin**	0	57	0	0	0	1	0	0	1	2	11	20	3	5	0.041
**Netilmicin**	0	58	0	3	0	1	5	2	7	11	3	9	0	1	0.033

Abbreviations: I, increased; N, no changed.

### 3.4 Correlation Between *mexY* Expression and MIC Reductionby Efflux Inhibitors

Curcumin decreased the MIC range in most aminoglycoside‐resistant isolates regardless of the mexY expression level. In addition, PAβN significantly reduced the aminoglycoside MIC in isolates with a ≥twofold overexpression of the mexY gene. The correlation between the mexY expression and the MIC reduction by efflux inhibitors is shown in Table 4.

**Table 4:** Relationship between the *mexY* gene expression and the MIC reduction by efflux inhibitors in aminoglycoside‐resistant *P. aeruginosa* isolates.

Inhibitors	Antibiotics	MIC reduction	No. (%) of *mexY* overproducer and nonoverproducer isolates				*p* value
			No change	2–3‐fold	4–9‐fold	> 10‐fold	
**PAβN**	Gentamicin	2–3‐fold (*n* = 12)	2 (16.66)	8 (66.66)	2 (16.66)	—	0.01
		> Tree‐fold (*n* = 3)	1 (33.33)	—	—	2 (66.66)	0.04
	Tobramycin	2–3‐fold (*n* = 18)	7 (38.88)	9 (50)	1 (5.55)	1 (5.55)	0.06
		> Tree‐fold (*n* = 2)	—	2 (100)	—	—	0.00
	Amikacin	2–3‐fold (*n* = 5)	1 (20)	3 (60)	1 (20)	—	0.00
		> Tree‐fold (*n* = 1)	—	1 (100)	—	—	0.00
	Kanamycin	2–3‐fold (*n* = 30)	15 (50)	14 (46.66)	1 (3.33)	—	0.05
		> Tree‐fold (*n* = 0)	—	—	—	—	—
	Streptomycin	2–3‐fold (*n* = 30)	16 (53.33)	12 (40)	1 (3.33)	1 (3.33)	0.06
		> Tree‐fold (*n* = 0)	—	—	—	—	—
	Spectinomycin	2–3‐fold (*n* = 37)	23 (62.16)	12 (32.43)	—	2 (5.40)	0.08
		> Tree‐fold (*n* = 3)	2 (66.66)	—	—	1 (33.33)	0.09
	Netilmicin	2–3‐fold (*n* = 29)	17 (58.62)	9 (31.03)	2 (6.89)	1 (3.44)	0.07
		> Tree‐fold (*n* = 4)	1 (25)	1 (25)	1 (25)	1 (25)	0.02
**Curcumin**	Gentamicin	2–3‐fold (*n* = 25)	17 (68)	7 (28)	1 (4)	—	0.09
		> Tree‐fold (*n* = 9)	5 (55.55)	1 (11.11)	2 (22.22)	1 (11.11)	0.07
	Tobramycin	2–3‐fold (*n* = 29)	19 (65.51)	7 (24.13)	2 (6.89)	1 (3.44)	0.09
		> Tree‐fold (*n* = 6)	3 (50)	2 (33.33)	—	1 (16.66)	0.05
	Amikacin	2–3‐fold (*n* = 3)	—	3 (100)	—	—	0.00
		> Tree‐fold (*n* = 3)	—	2 (66.66)	1 (33.33)	—	0.00
	Kanamycin	2–3‐fold (*n* = 38)	26 (68.42)	9 (23.68)	2 (5.26)	1 (2.63)	0.09
		> Tree‐fold (*n* = 4)	1 (25)	2 (50)	—	1 (25)	0.02
	Streptomycin	2–3‐fold (*n* = 28)	19 (67.85)	6 (21.42)	1 (3.57)	2 (7.14)	0.09
		> Tree‐fold (*n* = 11)	6 (54.54)	5 (45.45)	—	—	0.07
	Spectinomycin	2–3‐fold (*n* = 27)	17 (62.96)	10 (37.03)	—	—	0.08
		> Tree‐fold (*n* = 13)	8 (61.53)	3 (23.07)	—	2 (15.38)	0.08
	Netilmicin	2–3‐fold (*n* = 11)	7 (63.63)	3 (27.27)	—	1 (9.09)	0.08
		> Tree‐fold (*n* = 27)	16 (59.25)	8 (29.62)	2 (7.40)	1 (3.70)	0.07
**PAβN** + **Curcumin**	Gentamicin	2–3‐fold (*n* = 21)	14 (66.66)	7 (33.33)	—	—	0.09
		> Tree‐fold (*n* = 10)	5 (50)	2 (20)	2 (20)	1 (10)	0.05
	Tobramycin	2–3‐fold (*n* = 27)	18 (66.66)	7 (25.92)	1 (3.70)	1 (3.70)	0.09
		> Tree‐fold (*n* = 8)	4 (50)	2 (25)	1 (12.5)	1 (12.5)	0.05
	Amikacin	2–3‐fold (*n* = 8)	3 (37.5)	5 (62.5)	—	—	0.04
		> Tree‐fold (*n* = 1)	—	—	1 (100)	—	0.00
	Kanamycin	2–3‐fold (*n* = 32)	17 (53.12)	15 (46.87)	—	—	0.07
		> Tree‐fold (*n* = 0)	—	—	—	—	—
	Streptomycin	2–3‐fold (*n* = 31)	20 (64.51)	8 (25.80)	2 (6.45)	1 (3.22)	0.08
		> Tree‐fold (*n* = 6)	3 (50)	3 (50)	—	—	0.05
	Spectinomycin	2–3‐fold (*n* = 23)	17 (73.91)	5 (21.73)	—	1 (4.34)	0.09
		> Tree‐fold (*n* = 20)	11 (55)	7 (35)	—	2 (10)	0.06
	Netilmicin	2–3‐fold (*n* = 33)	20 (60.60)	9 (27.27)	2 (6.06)	2 (6.06)	0.07
		> Tree‐fold (*n* = 5)	3 (60)	2 (40)	—	—	0.07

## 4 Discussion

Aminoglycosides are important antibacterial drugs in the treatment of infections caused by *P. aeruginosa*, especially in patients with cystic fibrosis [10]. In the treatment of severe infections caused by this bacterium, the combination of aminoglycosides and beta‐lactams is used, while unfortunately, the resistance rate against these antibiotics is increasing [7]. A comparison of the antibiotic susceptibility patterns in the present study showed a relative high level aminoglycoside resistance compared to another Iranian research conducted in Tabriz [19]. These differences can be due to the different antibiotics prescriptions in hospitals, as amikacin resistance in our study was much lower than gentamicin and tobramycin due to the widespread use of gentamicin and tobramycin in this area.

The MIC results of the tested aminoglycosides in the present study showed that after the addition of inhibitors (curcumin and PAβN), the MIC of aminoglycosides decreased in 40‐100% of the aminoglycoside‐resistant isolates. Although curcumin and PAβN are not the specific MexXY inhibitors [26], but this efflux pump is able to export aminoglycosides. We found that curcumin reduced the MIC range in 100% of the isolates resistant to all aminoglycosides except amikacin and spectinomycin, suggesting efflux is the main mechanism responsible for aminoglycoside resistance. On the other hand, in a study conducted by Karaman et al., a decreased MIC range of tobramycin in combination with curcumin was observed [27]. Kali et al. also reported the synergistic effect of gentamicin and amikacin with curcumin against gram‐negative bacteria, including three isolates of *P. aeruginosa* [28]. These findings indicate that curcumin can probably inhibit the function of the MexXY‐OprM efflux pump, considering its role in decreasing aminoglycoside/aminocyclitol MIC range [9, 12]. Other studies reported an inhibitory effect of nano‐curcumin on biofilm formation and the transcription level of some virulence genes in *P. aeruginosa* [29, 30].

According to the MIC results, after adding PAβN, spectinomycin, netilmicin, and streptomycin showed the highest MIC reduction, while gentamicin and amikacin exhibited a 44.1% and 40% reduced MIC range (Table 2). Also, 100% of the tobramycin‐, netilmicin‐, and spectinomycin‐resistant isolates showed a decrease in the MIC range with a combination of both inhibitors. In the present study, the combination effect of curcumin and PAβN was better than the effect of PAβN alone and less than the effect of curcumin alone. These results suggest that curcumin alone was much more effective than PAβN in inhibiting the efflux pumps of the *P. aeruginosa* clinical isolates in the present study. This result was similar to a research conducted in India showing that 10% of the gentamicin‐resistant isolates were sensitive after treatment with curcumin (50 μg/mL), while PAβN had no effect [17]. Considering the similar effect of curcumin with the combination of two inhibitors, it can be concluded that it was curcumin that reduced the MICs of aminoglycosides. On the other hand, although PAβN is known as a nonspecific inhibitor against RND‐type multidrug efflux pumps, this inhibitor antagonizes the activity of aminoglycosides in a MexXY‐dependent manner [26]. Another study in Egypt reported that the synergistic effect of the combination of curcumin and PAβN on reducing the aminoglycosides MIC range against MDR *P. aeruginosa* isolates was much better than that of the curcumin or PAβN alone [16]. However, some studies have been performed based on the efflux pump inhibitors and their effect on decreasing the minimum inhibitory concentration of different antibiotics. Many of these studies have focused on enhancing antibiotics in combination with efflux pump inhibitors [8, 16, 17, 27, 28, 31]. We detected that curcumin was a better inhibitor considering the MIC reduction ability. The best effect of this inhibitor was observed in synergism by netilmicin, from which all resistant isolates showed a susceptibility or intermediate resistance phenotype. The same result was seen in a combination of streptomycin and curcumin, that 74.3% of the resistant isolates were susceptible to this aminoglycoside in synergism by curcumin. Although curcumin is a multitarget agent [32], aminoglycoside MIC reduction of *P. aeruginosa* with curcumin can be due to the MexXY inhibitory effect of curcumin.

On the other hand, PAβN was not a good marker for phenotypic detection of MexXY‐OprM efflux pump overexpression. Most aminoglycoside‐resistant isolates in the present study showed ineffective synergism by this inhibitor. Additionally, the aminoglycoside MIC reduction in combination with both efflux inhibitors tested was lower than that was seen by curcumin. We can conclude that this synergistic effect is related to curcumin. However, the aminoglycoside MIC reduction in the combination of curcumin and PAβN was better than curcumin alone for spectinomycin and amikacin. Considering the results obtained by other studies regarding the synergism effect of natural or chemical inhibitors with antibiotics, and adding the information related to our study, we can conclude that it may be possible to use these antimicrobial compounds in the near future. It will benefit the scientific community, if we overcome the antibiotic‐resistant organisms by more focuse on this inhibitors. Since the antibiotic resistance is the major health and treatment challenge in the world, and due to the less tendency of bacteria to develop resistance against natural compounds, further study on these compounds can be significant.

We detected that 45% of our isolates have overexpression of the mexY gene compared to the PAO1 control strain. Moreover, 14 isolates exhibited a more than threefold overexpression, while four isolates showed a ≥ 10‐fold overexpression of MexXY‐OprM efflux pump. However, Singh et al. in research conducted in Canada, showed that 13/15 isolates of *P. aeruginosa* collected from cystic fibrosis (CF) patients had a more than fourfold overexpression of the mexY gene ranging from 6.76‐ to 73‐fold compared to the PAO1 strain [2]. MexXY‐OprM overexpression can be due to the inhalation prescription of tobramycin and the environmental conditions that the organism encounters in CF lungs [2]. However, Dey et al. found a strong substrate preference of MexXY‐OprM for tobramycin and amikacin in clinical isolates of *P. aeruginosa* [12]. Evaluating the effect of PAβN and curcumin on the expression level of the MexXY‐OprM efflux pump is one of the limitations in this study. This assesment may be a significant scientific issue. If the inhibitors can reduce the expression of this efflux pump, we can count on these compounds to have a synergistic effect with antibiotics in the treatment of infections caused by this organism. Of course, the fact should be kept in mind that other mechanisms besides increasing the expression of the desired efflux pump (the production of drug‐modifying enzymes, upregulation of efflux pumps, bacterial RNA modification, and decreased drug influx) can also be effective in resistance to aminoglycosides and reduce the MIC of these drugs.

## 5 Conclusions

Efflux pump overexpression can increase the MIC ranges of antibiotics in clinical settings. This problem is a significant concern in the treatment procedure of hospitalized patients. Pseudomonas aeruginosa is an opportunistic pathogen that can use this mechanism for resistance to aminoglycosides. It seems that by inhibiting the function of these efflux systems, we can prevent the increase in antibiotics MIC ranges. We found that curcumin can be an effective efflux inhibitor that decreases the aminoglycosides MIC. Therefore, we may use this component as an adjuvant in combination with aminoglycosides for the treatment of patients infected by *P. aeruginosa* overexpressed MexXY‐OprM efflux pump. However, this idea needs an in vivo assessment.

## Author Contributions

Conceptualization: Hamid Reza Goli. Data curation: Hamid Reza Goli, Parisa Charkhi, Mohammad Reza Haghshenas, Bahman Mirzaei, Younes Khalili. Formal analysis: Parisa Charkhi, Bahman Mirzaei. Investigation: Hamid Reza Goli, Parisa Charkhi, Mohammad Reza Haghshenas, Younes Khalili. Methodology: Hamid Reza Goli, Parisa Charkhi, Bahman Mirzaei. Project administration: Hamid Reza Goli. Software: Hamid Reza Goli, Younes Khalili. Supervision: Hamid Reza Goli. Validation: Hamid Reza Goli. Visualization: Hamid Reza Goli, Parisa Charkhi, Mohammad Reza Haghshenas. Writing–original draft: Parisa Charkhi. Writing–review and editing: Hamid Reza Goli, Parisa Charkhi, Mohammad Reza Haghshenas, Bahman Mirzaei, Younes Khalili.

## Ethics Statement

This study was approved by the Iran National Committee for Ethics in Biomedical Research with the national ethical code IR. MAZUMS. REC.1397.368.

## Conflicts of Interest

The authors declare no conflicts of interest.

## Data Availability

All data produced or considered during this study are included in the published article.
